# The Role of Relationship Conflict for Momentary Loneliness and Affect in the Daily Lives of Older Couples

**DOI:** 10.1177/02654075221138022

**Published:** 2022-11-10

**Authors:** Elisa Weber, Gizem Hülür

**Affiliations:** 1 Department of Psychology and University Research Priority Program “Dynamics of Healthy Aging”, 27217 University of Zurich, Switzerland; 2 Department of Psychology, 9374 University of Bonn, Germany

**Keywords:** Conflict, loneliness, affect, experience sampling, dyadic analysis

## Abstract

**Background:** Intimate partner relationships foster individuals’ well-being throughout the lifespan. However, dissatisfying or conflict-laden relationships can have a detrimental impact on well-being and relationship quality. The majority of older adults live together with a spouse/partner, and intimate relationships are one of the most important social contexts in their daily lives. **Purpose:** Expanding on previous research, we examined the role of previous conflict on experiences of loneliness and affect in the daily lives of older partners from a dyadic perspective. Relationship duration and quality, personality traits (neuroticism and extraversion), conflict frequency during the measurement period, physical health as well as age were considered as moderators. **Study Sample and Data Analysis:** We used data from an experience sampling study with 151 older heterosexual couples (302 participants; 65+ years old) reporting on their positive and negative affect, loneliness, and previous experience of relationship conflict 6 times a day for 14 days. Data were analyzed using dyadic multilevel models.** Results:** For both men and women within couples, previous conflict was associated with an increased experience of negative affect and loneliness and a decreased experience of positive affect. Higher neuroticism predicted less positive and more negative affect following conflict for women and more loneliness for men. Higher relationship satisfaction predicted less increase in negative affect after conflict for female partners. Age, relationship duration, physical health, extraversion, and the number of conflict episodes showed no moderating effects. **Conclusions:** Our results support the notion that relationship conflict deteriorates emotional well-being in old age and renders older adults lonelier even in the context of intimate partner relationships.

## Introduction

Satisfying intimate partner relationships foster individual’s health and well-being (Kiecolt-Glaser & Wilson, 2017) and serve as a powerful protective factor against loneliness (Luhmann & Hawkley, 2016). On the other hand, dissatisfying or conflict-laden relationships may be detrimental for individual well-being and induce loneliness (Mund & Johnson, 2021). Intimate relationships are of considerable significance in old age, that is, when individuals perceive remaining life time as limited (Carstensen, 2006). However, while some attention has been given to examining affective reactions to relationship conflict in middle-aged couples (e.g., Almeida et al., 2002), these links have been understudied in old age. Against this background, the current paper examines the role of conflict on experiences of loneliness and affect in the daily lives of older intimate relationship partners as well as moderators of reactivity to conflict.

### Affect and Loneliness in Intimate Relationships

Empirical research has demonstrated that affective states are considerably shaped by one’s social environment. For example, intimate relationship partners reciprocally influence each other’s affective experiences through dynamic processes in daily life (Butler, 2011; Weber & Hülür, 2021). Similar to affect, the experience of loneliness is closely related to the social context, including the quantity and quality of social and intimate relationships. People experience loneliness when they fail to form close social bonds or perceive quantitative (e.g., social network size) or qualitative aspects (e.g., received social support) of their relationships as deficient or unsatisfactory (Holt-Lunstad et al., 2010). For example, research has shown that low relationship satisfaction and self-reported closeness are related to experiences of loneliness in couples (Mund et al., 2020).

### Couple Conflict and Partners’ Experiences of Affect and Loneliness

In the context of intimate relationships, conflict may arise when partners hold opposing opinions or interests or pursue goals which are incompatible (Bradbury et al., 2001; Kline et al., 2006). While some amount of conflict is normative and inevitable in intimate relationships, it is generally described as a highly stressful relational phenomenon (Feeney & Karantzas, 2017), which can lead to dissatisfaction or breakup when arguments are frequent or poorly managed (e.g., Clements et al., 2004; Kline et al., 2006). In line with this, couple conflict has been identified as a significant strain on middle-aged partners’ everyday affect, leading to less positive affective states (Almeida et al., 2002; Bolger et al., 1989) and elevated loneliness (Saenz, 2021) in husbands and wives.

#### Couple Conflict in Old Age

Couple conflict and conflict-related processes presumably change with advancing age. Socio-emotional selectivity theory (SST; e.g., Carstensen, 2006), a lifespan theory of motivation, posits that individuals increasingly prioritize emotion-related environments and close relationships when they perceive remaining lifetime to be limited. That is, older individuals may prefer social encounters with close others versus less close social contacts relative to younger adults for the purpose of nurturing positive emotions and reducing negative emotions. Moreover, SST suggests that older adults tend to process emotional information in a more positive light than younger adults. In line with this, empirical research has shown that relative to younger adults, older adults favor positive over negative information in attention and memory and appraise social situations as more positive and less distressing (age-related *positivity effect*; Charles & Carstensen, 2010; see also, Reed & Carstensen, 2012, for review). The SAVI model (*Strength and Vulnerability Integration*; Charles, 2010) builds upon SST by postulating age differences in (proactive) emotion regulation strategies that come with life experience (*strength*) as well as age-related difficulties in managing emotionally arousing situations, especially of negative valence (*vulnerability*). SAVI posits that older adults use their life experience to minimize or avoid negative experiences, e.g., escalation of conflict. A number of prior studies have examined predictions of SST and SAVI with respect to conflict in older couples. For example, older adults are likely to evaluate spousal interactions more positively than younger and middle-aged adults (Henry et al., 2007) and avoid conflict discussions whenever possible (Holley et al., 2013). Also, older adults are less likely than younger and middle-aged adults to report interpersonal tensions or stressors in daily life, or argue with spouses in response to tensions (Birditt et al., 2005). However, SAVI further suggests that older adults are less able than younger adults to downregulate negative emotional reactions when unable to avoid highly distressing experiences. That is, when older adults are unable to avoid or deescalate relationship conflict, high affective reactivity or experiences of loneliness may result. In line with this, spousal strain such as arguments has been found to engender negative emotional experiences (Carr et al., 2016; Choi & Marks, 2008) as well as loneliness (Chen & Feeley, 2014; Saenz, 2021) in old age. For example, Carr et al. (2016) observed that perceiving everyday marital strain, such as marital arguments, meaningfully increased momentary experiences of frustration, sadness and worry in older spouses.

#### Individual Moderators of Reactivity to Conflict

Within the framework of the vulnerability-stress-adaptation model, Bradbury and Karney (e.g., Bradbury and Karney, 2004) suggested that individual strengths and vulnerabilities should be considered to disentangle antecedents and relational effects of couple conflict. In line with this, empirical research has linked a multitude of individual and couple-level psychosocial factors to affective reactivity to couple conflict (e.g., Almeida et al., 2002).

##### Neuroticism

For example, neuroticism refers to a relatively stable tendency to experience unpleasant emotions in distressing or threatening situations (Verduyn & Brans, 2012) and a reduced tendency to downregulate negative emotions when unpleasant circumstances improve (Ng & Diener, 2009). Neuroticism predicts more loneliness cross-sectionally (Schermer & Martin, 2019) and increasing loneliness over time (Mund & Neyer, 2016). Importantly, higher levels of neuroticism are linked to lower relationship quality in partners (Lavee & Ben-Ari, 2004; Malouff et al., 2010). That is, individuals high in neuroticism may show negatively biased perceptions of partner interactions and dysfunctional emotion regulation strategies during conflict (Vater & Schröder–Abé, 2015). Moreover, individuals high in neuroticism are likely to be critical of their partners (McNulty, 2008), thus precipitating more frequent conflict (Bolger & Zuckerman, 1995; Finn et al., 2013).

##### Extraversion

In contrast to neuroticism, extraversion relates positively to the frequency, intensity and duration of positive emotions (Verduyn & Brans, 2012) as well as the stability of positive affect over time (Charles et al., 2001). Whereas loneliness and extraversion show moderately negative cross-sectional correlations (Schermer & Martin, 2019), findings on longitudinal associations are mixed (Mund & Neyer, 2016; von Soest, Luhmann, Hansen, & Gerstorf, 2020). Extraverted individuals are likely to respond to social stress with less physiological arousal and better adapt to recurrent stress (Lu & Wang, 2017), which might render them less susceptible to conflict and better at regulating their emotions during conflict (Vater & Schröder–Abé, 2015). In a daily diary study with 166 married middle-aged couples, Almeida et al. (2002) observed that affective reactivity to marital arguments in everyday life was lower in wives who were more extraverted than others.

#### Couple-level Moderators of Reactivity to Conflict

##### Relationship Duration

Moreover, areas of conflict likely change with relationship development (Kline et al., 2006). McGonagle et al. (1993) observed that couples married for a decade or longer experienced fewer, albeit more acrimonious marital disagreements than couples married for shorter time periods. In line with predictions of SAVI, couples may try to refrain from engaging in arguments with increasing age and relationship duration (Birditt et al., 2005; Holley et al., 2013) and escalate conflict only in moments of severe disagreement or when conflict cannot be avoided (Charles, 2010). Empirical findings have cemented this proposition by showing that long-term married couples in their 60s report less marital arguments across multiple possible conflict topics than couples in their 40s (e.g., money, leisure, sex, friends; Haase & Shiota, 2019; Levenson et al., 1993). In addition, McGonagle et al. (1993) suggested that increasing relationship duration has couples develop a sense of confidence that conflict can be weathered and evaluate it as less threatening to terminate the relationship. It may be expected that increasing relationship duration buffers the detrimental impact of conflict on partners’ daily experiences of affect and loneliness.

##### Relationship Satisfaction

On the contrary, low relationship satisfaction may exacerbate the detrimental influence of conflict. Even though partner relationships serve as a powerful protective factor against loneliness (Luhmann & Hawkley, 2016), they may increase loneliness when experienced as unsatisfying or of otherwise low quality (Mund et al., 2020). Perceived negative relationship quality, such as being overly critical or demanding, has been found to influence both one’s own and one’s partner’s loneliness (Moorman, 2016). Moreover, both own and partner’s relationship dissatisfaction may predict emotional distress in men and women (Røsand et al., 2012).

##### Conflict Frequency

Engaging to more frequent conflict may lower relationship satisfaction and increase loneliness as well as perceived relationship instability of intimate partners over time (Johnson et al., 2018; Mund et al., 2020). Higher frequency of marital arguments is linked to stronger affective reactions to each single argument (Almeida et al., 2003; Jacobson et al., 1982). However, evidence on the links between the frequency of relationship conflict and affective reactions to each single argument remains scarce and inconclusive. Generally, older couples report lower numbers of marital arguments across multiple possible conflict topics than middle-aged couples (Haase & Shiota, 2019; Levenson et al., 1993). In line with this finding, Holley et al. (2013) observed that spouses’ avoidance behaviors during conflict increased linearly with age, in that older spouses actively avoided conflict discussions, for example, by changing the topic or diverting attention. This may reflect an adaptive strategy of moving away from toxic conflict areas in old age, or a mutual acceptance that certain topics are unlikely to undermine the relationship and thus unworthy of conflict. Nonetheless, higher conflict frequency may both emanate from and create a stressful and potentially harmful marital environment hindering constructive responses to conflict (Neff & Karney, 2017). In line with this, Almeida et al. (2002) showed that husbands’ emotional reactivity to marital arguments was higher in couples with more frequent arguments. It is possible that partners find it harder to return to baseline affective arousal after conflict when exposed to constant and chronic relationship distress (e.g., Almeida et al., 2002).

### The Present Study

The present study expands on prior research by examining the role of previous conflict on everyday experiences of loneliness and affect in older couples from a dyadic perspective. Prior research on daily relationship conflict has largely involved couples in middle adulthood (Almeida et al., 2002; Bolger et al., 1989) and conflict was assessed retrospectively and based on less frequent observations, i.e., one measurement at the end of the day. Moreover and to the best of our knowledge, our study is the first to address the impact of conflict on everyday loneliness in old age, which is highly relevant since frequent experiences of loneliness pose a significant risk factor for morbidity and mortality (Holt-Lunstad et al., 2010). Furthermore, studying older couples is important as they may experience intimate relationships and emotions evoked by partner interactions differently than younger and middle-aged couples (e.g., Carstensen, 2006; Charles, 2010). Based on prior studies (Almeida et al., 2002; Bolger et al., 1989), we hypothesized that conflict is associated with decrease in positive and increase in negative affect and loneliness. In order to understand individual and couple-level resources and risk factors, we considered personality traits (neuroticism and extraversion), relationship duration and quality, as well as frequency of conflict during the measurement period as potential moderators. Specifically, we expected higher neuroticism and higher conflict frequency to be linked with higher conflict reactivity, and higher extraversion, relationship satisfaction and duration relating to lower conflict reactivity. We controlled for individual differences in age and health.

## Method

We used data from a daily-life experience sampling study with older couples in Switzerland and Southern Germany (Weber & Hülür, 2021). Participants completed up to 84 reports (six times per day for 14 consecutive days) about their momentary affect, momentary loneliness and previous conflict. Data were collected via iPads provided to both partners for 14 days. The research protocol was reviewed by the Ethics Committee of the Faculty of Arts and Social Sciences at the University of Zurich in Switzerland (2018.4.4).

### Participants

Participants were recruited via announcements in local newspapers and screened according to the following criteria: (a) both partners were age 65+ years old, (b) partners were living together (cohabiting or married), (c) participants could see and hear sufficiently well (experience sampling questionnaires were prompted by auditory signals) and (d) participants had sufficient German proficiency to answer questionnaires independently. As analyses were based on models for distinguishable dyads with gender as the distinguishing characteristic, we had to exclude data from one homosexual couple. We had to exclude data from one additional couple because one or both partners had missing data on moderator variables. Finally, data from 151 heterosexual couples were included in our analyses.

Participants were on average 73 years old (*SD* = 5; range = 65–86); 89% were married and 11% were in a cohabiting relationship for an average of 40 years (range = 1 year to 64 years). 164 participants (54%) had a school degree that qualified them for tertiary education (at a university or a university of applied science) and 110 participants (36%) had participated in or completed tertiary education at a university or a university of applied science. 198 participants (66%) were retired and not working. 50 participants (17%) reported to be pensioners yet still working part-time. 46 participants (15%) reported having never worked and receiving no pension paid by the state. Seven participants (2%) were still working and not yet retired. One participant gave no information on his/her work or retirement status. In 102 couples (68%), both partners were not working (never worked or retired). In 6 couples (4%), both partners were retired yet still working part-time. In 42 couples (28%), one partner was retired or has never worked while the other partner was still working part-time. In 1 couple, one of the partners gave no information on his/her work or retirement status. Upon recruitment, we made sure that partners were able to spend the majority of their daytime (i.e., between 9 a.m. and 9 p.m.) together. Participants noted that their partners were present (within visibility range) during 67% of all measurement occasions.

### Procedure

Each couple attended an introductory session at the University of Zurich or at their place of residence, depending on preference. The 14-day study period was scheduled for 2 weeks which couples considered typical, i.e., in which they would go about their normal daily routines. If unusual events occurred, such as unplanned hospital visits, we rescheduled the study period. Trained research assistants conducted introductory sessions to provide detailed instruction on study procedures and hand an iPad to each participant. In addition, couples were given background questionnaires (online or paper-pencil, depending on preference) to complete during the 14-day study period.

Over the next 14 days, participants completed six short questionnaires per day which were prompted by an iOS-based application (iDialogPad; G. Mutz, Cologne, Germany). Questionnaires were sent in a pseudo-randomized sequence between 9 a.m. and 9 p.m. Each questionnaire was scheduled within a time window of 2 hours. There was a minimum of a one-hour interval between two questionnaires. Participants could choose to delay their response up to 4 times for 5–15 minutes. Audio signal announcing questionnaires activated simultaneously for both partners, and each couple followed their own questionnaire schedule. Participants were instructed to answer incoming questionnaires both at home and outside and to refrain from discussing responses with partners.

Participants completed 89% of all experience sampling questionnaires (range 42%–100%). 10.8% completed all 84 experience sampling questionnaires. Completion rates in ESM studies typically vary around 70%–80% of all possible reports (Rintala et al., 2019; Sun et al., 2021). Thus, participants in our study exhibited relatively high completion rates. Data in our study are missing at random and did not systematically differ across key study variables. The number of missing values per person did not correlate significantly with gender (*r* = .03, *p* = .608), conflict frequency (*r* = −.05, *p* = .400), extraversion (*r* = .02, *p* = .680), neuroticism (*r* = .05, *p* = .360), physical health (*r* = −.03, *p* = .567), relationship satisfaction (*r* = −.003, *p* = .960), or relationship duration (*r* = .01, *p* = .863). However, the number of missing values per person was significantly correlated with age (*r* = .21, *p* = < .01). That is, older participants were more likely to miss out on single iPad questionnaires.

On day 2 of the study, a research assistant contacted couples by phone to answer potential questions. After the 14-day study, a post-test session was scheduled in which couples had the chance to give feedback on the study. Post-test sessions were conducted by research assistants at the laboratory or at couples’ homes.

### Measures

We made use of repeated reports of momentary loneliness, positive and negative affect at each measurement point as well as conflict occurring since the last measurement point as time-varying variables. Items indicating momentary loneliness, positive and negative affect were assessed with a sliding scale ranging from 0% (not at all) to 100% (very much).

#### Positive and Negative Affect

Participants used the sliding scale to answer the question: “How do you feel in this moment?” for the items happy, inspired, content, and calm (positive affect) and for the items sad, nervous, anxious, and upset (negative affect). Responses were averaged across four items, respectively. We estimated within-person reliability of positive affect in a multilevel confirmatory factor analysis (Bolger & Laurenceau, 2013) in MPlus (Muthén & Muthén, 1998–2017), which resulted in *omega* = 0.78 for positive affect and *omega* = 0.68 for negative affect.

#### Loneliness

Participants answered the question: “How lonely you feel in this moment?” using the sliding scale.

#### Conflict

Participants indicated whether they experienced a disagreement, argument, or conflict with their partner since the last measurement occasion (0 = no; 1 = yes).

#### Between-Person and Between-Couple Difference Characteristics

Participants’ *gender* was indicated by a binary variable (0 = male, 1 = female) and used as the distinguishing characteristic in dyadic analyses. *Neuroticism* and *extraversion* were assessed using the Big Five Inventory (BFI; John & Srivastava, 1999; German version: Lang et al., 2001) with scores from 1 (low) to 5 (high). *Relationship satisfaction* was assessed using the Relationship Assessment Scale (RAS; Hendrick, 1988; German version: Sander & Böcker, 1993) with scores from 1 (low) to 5 (high). *Relationship duration* indicated the number of years partners were in a cohabiting relationship or married. *Conflict frequency* indicated the number of conflicts that occurred between partners across the 14-day experience sampling period (mean = 4.4; range = 0–35). Participants’ *age* was scaled in years. *Physical health* was assessed using the Physical Functioning scale from the Short-Form-36 Health Survey (SF-36; Ware & Sherbourne, 1992; German version: Bullinger et al., 1995) with scores ranging from 0 (poor health) to 100 (excellent health). All predictors were centered at sample means. Table 1 shows descriptive statistics and correlations of study variables.

**Table 1.** Descriptive Statistics and Correlations of Study Variables.

		*M (SD)*	Loneliness		Positive Affect		Negative Affect		Age		Duration		Satisfaction		Health		Neuroticism		Extraversion		Conflict Frequency	
			w	m	w	m	w	m	w	m	w	m	w	m	w	m	w	m	w	m	w	m
Loneliness	w	13.80 (14.76)																				
	m	13.22 (11.64)																				
Positive affect	w	71.73 (12.63)	**−0.45**	**−0.40**																		
	m	73.41 (11.62)		**−0.45**																		
Negative affect	w	17.82 (12.72)	**0.66**	**0.43**	**−0.59**	**−0.34**																
	m	16.10 (11.96)		**0.76**		**−0.62**																
Age	w	71.57 (5.03)	**0.19**	**0.20**	**−0.19**	**−0.29**	**0.26**	**0.27**														
	m	73.61 (4.91)		0.16		**−0.25**		**0.17**														
Duration	w	40.22 (15.43)	−0.04	−0.03	−0.04	−0.08	0.00	0.09	**0.19**	**0.27**												
	m	40.22 (15.43)		−0.03		−0.08		0.09		**0.27**												
Satisfaction	w	3.96 (0.86)	**−0.37**	**−0.31**	**0.26**	**0.21**	**−0.31**	**−0.27**	**−0.19**	**−0.19**	0.03	0.03										
	m	4.01 (0.77)		**−0.18**		**0.29**		**−0.21**		−0.06		−0.02										
Health	w	82.22 (18.85)	**−0.33**	−0.11	**0.18**	0.00	**−0.29**	−0.15	**−0.28**	**−0.27**	−0.06	−0.06	0.13	0.13								
	m	86.72 (15.00)		−0.13		0.02		−0.08		**−0.23**		−0.16		0.09								
Neuroticism	w	2.61 (0.66)	**0.24**	0.10	**−0.35**	−0.14	**0.39**	**0.20**	**0.18**	0.06	0.03	0.03	**−0.25**	−0.27	**−0.18**	0.06						
	m	2.34 (0.52)		**0.24**		**−0.25**		**0.32**		0.06		0.03		−0.09		−0.05						
Extraversion	w	3.60 (0.64)	−0.11	−0.02	0.15	**0.17**	−0.12	−0.07	−0.14	−0.08	−0.09	−0.09	0.11	0.09	0.10	0.04	**−0.24**	0.07				
	m	3.29 (0.68)		−0.13		**0.27**		**−0.23**		−0.05		−0.09		0.12		0.03		**−0.39**				
Conflict frequency	w	4.25 (4.46)	0.13	**0.28**	**−0.38**	**−0.26**	**0.28**	**0.29**	−0.01	−0.03	0.08	0.08	−0.15	−0.11	−0.09	−0.09	**0.24**	0.07	0.23	0.01		
	m	4.46 (5.61)		**0.23**		**−0.19**		**0.34**		0.00		0.10		−0.22		−0.14		0.09		−0.02		

*Notes. n* = 151 couples*. w* = women, *m* = men. Variables averaged across all observations per participant. *M* = mean; *SD* = standard deviation. Loneliness and affect from 0-100%; age in years; duration = relationship duration in years; satisfaction = relationship satisfaction from 1 to 5; health = physical health from 0 to 100 (higher values = better health); neuroticism from 1 to 5; extraversion from 1 to 5; conflict frequency = number of conflicts.

Significant *p*-values (<.05) in bold.

### Data Analysis

We applied longitudinal multilevel modeling for distinguishable dyads to address statistical dependence in longitudinal dyadic data (Hülür & Weber, 2019). Models were set up with two levels (between and within dyads, Bolger & Laurenceau, 2013), with gender as the distinguishing variable. Models were estimated separately for loneliness, positive and negative affect. In each model, the outcome in relation to presence of conflict since the last measurement point was modelled as a two-equation multivariate system, with one equation referring to women and the other equation referring to men. The baseline level 1 models for loneliness (1), positive (2) and negative affect (3) of women (subscript *w*) were specified as

(1) $${\text{Loneliness}}_{\text{ti}w}{=ß}_{0iw}{+ß}_{1iw}\left({\text{conflict}}_{\text{ti}w}\right){+e}_{\text{ti}w}$$

(2) $${\text{Positive}\;\text{affect}}_{\text{ti}w}{=ß}_{0iw}{+ß}_{1iw}\left({\text{conflict}}_{\text{ti}w}\right){+e}_{\text{ti}w}$$

(3) $$\text{Negative}\;{\text{affect}}_{\text{ti}w}{=ß}_{0iw}{+ß}_{1iw}\left({\text{conflict}}_{\text{ti}w}\right){+e}_{\text{ti}w}$$

(4) $${ß}_{0iw}=\gamma_{00w}{+u}_{0iw}$$

(5) $${ß}_{1iw}=\gamma_{10w}{+u}_{1iw}$$

where loneliness and affect of person *i* at time *t* was a function of a person-specific intercept coefficient, ${ß}_{0iw}$ , a person-specific coefficient, ${ß}_{1iw}$ , indicating the extent to which person *i*’s loneliness and affect differed depending on whether conflict had occurred since the previous occasion, and residual error, which correlated within dyads. An equivalent model was specified for men. *u*s reflected individual-specific deviations from ß parameters, which correlated for women and men. _u0i*w*_ indicated individual differences in baseline affect and loneliness, respectively. _u1i*w*_ indicated individual differences in conflict reactivity.

In a next step, individual (neuroticism, extraversion) and couple-level predictors (relationship satisfaction, relationship duration, conflict frequency) as well as control variables (age, health status) were included. Between-person differences in ß coefficients were modelled, for women, as

(6) $$\begin{array}{l} {ß}_{0iw}=\gamma_{00w}+\gamma_{01w}\left({\text{age}}_{iw}\right)+\gamma_{02w}\left(\text{relationship}\;{\text{satisfaction}}_{iw}\right)+\gamma_{03w}\left({\text{physical}\;\text{health}}_{iw}\right)+\gamma_{04w}\left({\text{neuroticism}}_{iw}\right)+\gamma_{05w}\left({\text{extraversion}}_{iw}\right)+\\ {ß}_{06w}\left(\text{conflict}\;{\text{frequency}}_{iw}\right)+\gamma_{07w}\left(\text{relationship}\;{\text{duration}}_{iw}\right){+u}_{0iw} \end{array}$$

(7) $$\begin{array}{l} {ß}_{1iw}=\gamma_{10w}+\gamma_{11w}\left({\text{age}}_{iw}\right)+\gamma_{12w}\left(\text{relationship}\;{\text{satisfaction}}_{iw}\right)+\gamma_{13w}\left({\text{physical}\;\text{health}}_{iw}\right)+\gamma_{14w}\left({\text{neuroticism}}_{iw}\right){+1}_{05w}\left({\text{extraversion}}_{iw}\right)+\\ \gamma_{16w}\left(\text{conflict}\;{\text{frequency}}_{iw}\right)+\gamma_{17w}\left(\text{relationship}\;{\text{duration}}_{iw}\right){+u}_{1iw} \end{array}$$

where γ _00*w*_ and γ _10*w*_ indicated the sample-average level of loneliness and affect as well as differences in these variables depending on conflict for the typical women. The other γ parameters indicated the extent to which ß parameters were related to individuals relationship satisfaction, neuroticism, extraversion, conflict frequency, relationship duration, age and physical health. *u*s reflected individual-specific deviations from ß parameters, which were allowed to correlate for women and men. An equivalent model was specified for men.

Models were estimated with restricted maximum likelihood in *lme4* (Bates et al., 2015) in *R* (R Core Team, 2013). Bootstrap resampling was applied to obtain bootstrapped regression coefficients and standard errors of fixed effects based on 1000 iterations, which were used to compute *p*-values. Statistical significance was evaluated at *p* < .05.

## Results

Descriptive statistics and correlations of study variables are displayed in Table 1. Descriptive statistics and correlations of time-varying variables (loneliness and affect) are based on the person-mean of repeated measures. Regarding these correlations, loneliness and negative affect showed positive correlations with age, neuroticism and conflict frequency, whereas positive affect decreased with increasing values on these variables. Moreover, loneliness and negative affect yielded negative correlations with health and relationship satisfaction, whereas positive affect showed the opposite pattern.

### Loneliness

Table 2 shows the results from our baseline model examining the effects of previous conflict on loneliness. Table 3 shows the results from the model including moderators of conflict reactivity and control variables. The effects of conflict on loneliness showed the same pattern in both sets of analyses. Below, we summarize findings from Table 3.

**Table 2.** The Role of Conflict on Experiences of Loneliness (Baseline).

Fixed Effects	Women		Men	
Predictor	γ	SE	γ	SE
Intercept	**13.79**	1.16	**13.17**	0.93
Conflict	**6.13**	1.24	**6.57**	1.33

Random Effects	Variance (*σ*^*2*^)	Correlation			
		(1)	(2)	(3)	(4)
Women:Intercept (u_0*iw*_) (1)	211.9				
Men:Intercept (u_0*im*_) (2)	128.1	0.58			
Women: Conflict (u_1*iw*_) (3)	110.7	0.17	0.09		
Men:Conflict (u_1*im*_) (4)	118.9	0.03	0.02	0.47	
Residual	270.1				
AIC	192305.6				
BIC	192426.0				

*Notes. n* = 151 couples*. w* = women, *m* = men. γ = regression coefficient; SE = standard error; u = random effect; *σ*^*2*^ = variance. Fixed effects = regression coefficients and standard errors are presented. Random effects = variances and correlations are presented. Outcome variable: loneliness on a scale from 0% (not at all) to 100% (very much). Statistical significance evaluated at *p* < .05.

Significant *p*-values in bold.

**Table 3.** The Role of Conflict on Experiences of Loneliness (including Moderators).

Fixed Effects	Women		Men	
Predictor	γ	SE	γ	SE
Intercept	**12.85**	1.15	**13.77**	0.99
Conflict	**5.82**	1.60	**7.56**	1.60
Age	0.02	0.22	**0.37**	0.18
Relationship duration	−0.05	0.07	−0.08	0.06
Relationship satisfaction	**−4.03**	1.14	−1.02	1.05
Physical health	**−0.22**	0.05	−0.06	0.05
Neuroticism	2.48	1.58	**3.41**	1.67
Extraversion	−0.61	1.58	−0.36	1.27
Conflict frequency	−0.24	0.23	0.22	0.15
Conflict*Age	0.08	0.29	0.39	0.25
Conflict*Duration	0.00	0.09	−0.07	0.09
Conflict*Satisfaction	−1.48	1.49	2.43	1.61
Conflict*Physical health	−0.07	0.07	0.09	0.10
Conflict*Neuroticism	1.38	2.23	**5.58**	2.76
Conflict*Extraversion	-–0.72	2.24	0.71	1.95
Conflict*Frequency	−0.04	0.30	0.04	0.21

Random Effects	**Variance** (*σ*^*2*^)	Correlation			
		(1)	(2)	(3)	(4)
Women:Intercept (u_0*iw*_) (1)	172.0				
Men:Intercept (u_0*im*_) (2)	117.2	0.53			
Women: Conflict (u_1*iw*_) (3)	121.0	0.04	0.01		
Men:Conflict (u_1*im*_) (4)	106.6	−0.03	0.00	0.46	
Residual	267.2				
AIC	190155.4				
BIC	190500.2				

*Notes. n* = 151 couples*. w* = women, *m* = men. γ = regression coefficient; SE = standard error; u = random effect; *σ*^*2*^ = variance. Fixed effects = regression coefficients and standard errors are presented. Random effects = variances and correlations are presented. Outcome variable: loneliness on a scale from 0% (not at all) to 100% (very much); age in years; duration = relationship duration in years; satisfaction = relationship satisfaction on a scale from 1 to 5 (higher values indicate higher satisfaction); health = physical health on a scale from 0 to 100 (higher values indicate better health); neuroticism on a scale from 1 to 5 (higher values indicate higher neuroticism); extraversion on a scale from 1 to 5 (higher values indicate higher extraversion); conflict frequency = number of conflicts that occurred between partners. *Statistical* significance evaluated at *p* < .05.

Significant *p*-values in bold.

On average, women rated their momentary loneliness at γ _00*w*_ = 12.85 points and men rated their momentary loneliness at γ _00*m*_ = 13.77 points from 0 to 100. Conflict since the previous measurement occasion was associated with significantly higher loneliness for female ( γ _10*w*_ = 5.82) and male ( γ _10*m*_ = 7.56) partners. Higher baseline loneliness was linked to older age (γ_01*m*_ = 0.37) and higher levels of neuroticism ( γ _04*m*_ = 3.41) in men as well as lower levels of relationship satisfaction ( γ _02*w*_ = −4.03) and physical health ( γ _03*w*_ = −0.22) in women. Moreover, higher neuroticism predicted more reactivity to relationship conflict for loneliness in men ( γ _14*m*_ = 5.58). That is, increases in loneliness following conflict were higher in men with higher levels of neuroticism.

Residual random variance around intercepts amounted to u_0*iw*_ = 172.0 for women and u_0*im*_ = 117.2 for men, indicating individual differences in levels of loneliness after accounting for the variables included in our model. Residual random variance around average effects of conflict on loneliness amounted to u_1*iw*_ = 121.0 for women and u_1*im*_ = 106.6 for men, indicating individual differences in conflict reactivity. Random intercepts of male and female loneliness correlated at *r* = .53, that is, women with higher than average overall loneliness likely had a partner with higher than average overall loneliness. Random effects of conflict on male and female loneliness correlated at *r* = .46, that is, women who were more reactive to conflict than average likely had a partner who was also more reactive.

**Table 4.** The Role of Conflict on Experiences of Positive Affect (Baseline).

Fixed Effects	Women		Men	
Predictor	γ	SE	γ	SE
Intercept	**71.70**	1.00	**73.38**	0.94
Conflict	**−11.31**	1.15	**−9.38**	0.87

Random Effects	Variance (*σ*^*2*^)	Correlation			
		(1)	(2)	(3)	(4)
Women:Intercept (u_0*iw*_) (1)	151.1				
Men:Intercept (u_0*im*_) (2)	131.5	0.42			
Women: Conflict (u_1*iw*_) (3)	122.3	−0.15	−0.08		
Men:Conflict (u_1*im*_) (4)	58.9	−0.17	−0.01	0.46	
Residual	114.7				
AIC	173230.1				
BIC	173350.5				

*Notes. n* = 151 couples*. w* = women, *m* = men. γ = regression coefficient; SE = standard error; u = random effect; *σ*^*2*^ = variance. Fixed effects = regression coefficients and standard errors are presented. Random effects = variances and correlations are presented. Outcome variable: positive affect on a scale from 0% (not at all) to 100% (very much). *Statistical* significance evaluated at *p* < 0.05. Significant *p*-values in bold.

### Positive Affect

Table 4 shows the results from our baseline model examining the effect of previous conflict on positive affect. Table 5 summarizes the results from our model including moderators and control variables. Below, we summarize findings from Table 5. On average, momentary positive affect was rated at γ_00*w*_ = 72.29 points for women and γ_00*m*_ = 73.50 points for men. from 0 to 100. Experiencing conflict since the previous occasion was associated with significantly lower positive affect both for female (γ_10*w*_ = −10.74) and male (γ_10*m*_ = −10.28) partners. Women and men higher in relationship satisfaction (γ_02*w*_ = 2.24; γ_02*m*_ = 3.50) and lower in neuroticism (γ_04*w*_ = −3.57; γ_04*m*_ = −3.84) reported more positive affect than women and men lower in relationship satisfaction and higher in neuroticism, respectively. Men higher in extraversion reported higher positive affect than men lower in extraversion (γ_05*m*_ = 3.13). Women who experienced conflict more frequently reported lower positive affect on average (γ_06*w*_ = −0.58). Neuroticism moderated reactivity to conflict in women, with women higher in neuroticism showing more decline in positive affect following conflict than women lower in neuroticism (γ_14*w*_ = −4.86).

**Table 5.** The Role of Conflict on Experiences of Positive Affect (including Moderators).

Fixed Effects	Women		Men	
Predictor	γ	SE	γ	SE
Intercept	**72.29**	0.98	**73.50**	0.96
Conflict	**−10.74**	1.40	**−10.28**	1.13
Age	−0.19	0.19	**−0.66**	0.18
Relationship duration	0.01	0.06	0.01	0.06
Relationship satisfaction	**2.24**	1.09	**3.50**	1.05
Physical health	0.08	0.05	−0.09	0.05
Neuroticism	**−3.57**	1.50	**−3.84**	1.54
Extraversion	0.71	1.37	**3.13**	1.22
Conflict frequency	**−0.58**	0.21	−0.19	0.15
Conflict*Age	−0.44	0.24	−0.10	0.19
Conflict*Duration	−0.01	0.08	−0.06	0.07
Conflict*Satisfaction	0.66	1.27	−0.35	1.15
Conflict*Physical health	−0.01	0.06	−0.07	0.06
Conflict*Neuroticism	**−4.86**	1.82	−2.66	1.99
Conflict*Extraversion	1.36	1.78	−1.76	1.35
Conflict*Frequency	−0.08	0.25	0.18	0.15

Random Effects	Variance (*σ*^*2*^)	Correlation			
		(1)	(2)	(3)	(4)
Women:Intercept (u_0*iw*_) (1)	122.96				
Men:Intercept (u_0*im*_) (2)	107.75	0.34			
Women: Conflict (u_1*iw*_) (3)	131.95	−0.31	−0.47		
Men:Conflict (u_1*im*_) (4)	56.42	−0.13	−0.06	0.45	
Residual	113.68				
AIC	171372.9				
BIC	171717.6				

*Notes. n* = 151 couples*. w* = women, *m* = men. γ = regression coefficient; SE = standard error; u = random effect; *σ*^*2*^ = variance. Fixed effects = regression coefficients and standard errors are presented. Random effects = variances and correlations are presented. Outcome variable: positive affect on a scale from 0% (not at all) to 100% (very much); age in years; duration = relationship duration in years; satisfaction = relationship satisfaction on a scale from 1 to 5 (higher values indicate higher satisfaction); health = physical health on a scale from 0 to 100 (higher values indicate better health); neuroticism on a scale from 1 to 5 (higher values indicate higher neuroticism); extraversion on a scale from 1 to 5 (higher values indicate higher extraversion); conflict frequency = number of conflicts that occurred between partners. *Statistical* significance evaluated at *p* < .05.

Significant *p*-values in bold.

Residual random variance around intercepts amounted to u_0*iw*_ = 122.96 for women and u_0*im*_ = 107.75 for men. Residual random variance around average effects of conflict on positive affect amounted to u_1*iw*_ = 131.95 for women and u_1*im*_ = 56.42 for men. Women with higher than average overall positive affect likely had a partner with higher than average overall positive affect (*r* = .34). Women who were more reactive to conflict in terms of positive affect likely had a partner who was more reactive to conflict (*r* = .45).

**Table 6.** The Role of Conflict on Experiences of Negative Affect (Baseline).

Fixed Effects	Women		Men	
Predictor	γ	SE	γ	SE
Intercept	**17.82**	1.03	**16.06**	0.95
Conflict	**10.20**	1.10	**8.91**	1.00

Random Effects	Variance (*σ*^*2*^)	Correlation			
		(1)	(2)	(3)	(4)
Women:Intercept (u_0*iw*_) (1)	156.04				
Men:Intercept (u_0*im*_) (2)	136.14	0.48			
Women: Conflict (u_1*iw*_) (3)	104.40	−0.10	−0.07		
Men:Conflict (u_1*im*_) (4)	78.78	−0.01	0.12	0.38	
Residual	118.85				
AIC	174039.5				
BIC	174159.9				

*Notes. n* = 151 couples*. w* = women, *m* = men. γ = regression coefficient; SE = standard error; u = random effect; *σ*^*2*^ = variance. Fixed effects = regression coefficients and standard errors are presented. Random effects = variances and correlations are presented. Outcome variable: negative affect on a scale from 0% (not at all) to 100% (very much). *Statistical* significance evaluated at *p* < .05.

Significant *p*-values in bold.

### Negative Affect

Table 6 summarizes the results from our baseline model on effects of previous conflict on negative affect. Table 7 displays the results from the model including moderators and control variables. Below, we summarize findings from Table 7.  On average, women rated their momentary negative affect at γ_00*w*_ = 16.89 points. Men rated their momentary negative affect at γ_00*m*_ = 16.57 points from 0 to 100. Experiencing conflict since the previous occasion was associated with increase in negative affect both for women (γ_10*w*_ = 9.75) and men (γ_10*m*_ = 9.63). Women with higher relationship satisfaction (γ_02*w*_ = −2.80), lower neuroticism (γ_04*w*_ = 4.20), and better physical health (γ_03*w*_ = −0.13) reported less negative affect. Men high in neuroticism (γ_04*m*_ = 5.55) and conflict frequency (γ_06*m*_ = 0.56) reported more negative affect. Women with higher relationship satisfaction showed less reactivity to conflict, i.e., the increase in negative affect following conflict (γ_12*w*_ = −2.59) was lower. Women higher in neuroticism showed more reactivity to conflict, that is, increase in negative affect was higher (γ_14*w*_ = 4.79).

**Table 7.** The Role of Conflict on Experiences of Negative Affect (Including Moderators).

Fixed Effects	Women		Men	
Predictor	γ	SE	γ	SE
Intercept	**16.89**	0.97	**16.57**	0.93
Conflict	**9.75**	1.27	**9.63**	1.19
Age	0.17	0.20	0.33	0.18
Relationship duration	−0.04	0.06	0.01	0.06
Relationship satisfaction	**−2.80**	1.07	−1.68	1.11
Physical health	**−0.13**	0.05	0.03	0.05
Neuroticism	**4.20**	1.46	**5.55**	1.74
Extraversion	−0.24	1.37	−1.78	1.28
Conflict frequency	0.31	1.20	**0.56**	0.15
Conflict*Age	0.08	0.23	0.16	0.22
Conflict*Duration	0.12	0.07	−0.04	0.07
Conflict*Satisfaction	**−2.59**	1.24	1.18	1.34
Conflict*Physical health	0.00	0.05	0.10	0.07
Conflict*Neuroticism	**4.79**	1.76	4.20	2.26
Conflict*Extraversion	−0.63	1.67	−0.71	1.59
Conflict*Frequency	−1.19	0.24	−0.01	0.18

Random Effects	Variance (*σ*^*2*^)	Correlation			
		(1)	(2)	(3)	(4)
Women:Intercept (u_0*iw*_) (1)	117.90				
Men:Intercept (u_0*im*_) (2)	111.56	0.37			
Women: Conflict (u_1*iw*_) (3)	95.29	−0.27	−0.34		
Men:Conflict (u_1*im*_) (4)	75.99	−0.06	0.05	0.31	
Residual	117.19				
AIC	172166.7				
BIC	172511.5				

*Notes. n* = 151 couples*. w* = women, *m* = men. γ = regression coefficient; SE = standard error; u = random effect; *σ*^*2*^ = variance. Fixed effects = regression coefficients and standard errors are presented. Random effects = variances and correlations are presented. Outcome variable: negative affect on a scale from 0% (not at all) to 100% (very much); age in years; duration = relationship duration in years; satisfaction = relationship satisfaction on a scale from 1 to 5 (higher values indicate higher satisfaction); health = physical health on a scale from 0 to 100 (higher values indicate better health); neuroticism on a scale from 1 to 5 (higher values indicate higher neuroticism); extraversion on a scale from 1 to 5 (higher values indicate higher extraversion); conflict frequency = number of conflicts that occurred between partners. *Statistical* significance evaluated at *p* < .05.

Significant *p*-values in bold.

Residual random variance around intercepts amounted to u_0*iw*_ = 117.90 for women and u_0*im*_ = 111.56 for men. Residual random variance around reactivity to conflict amounted to u_1*iw*_ = 95.29 for women and u_1*im*_ = 75.99 for men. Women with higher than average overall negative affect likely had a partner reporting higher than average overall negative affect (*r *= .37). Women who were more reactive to conflict in terms of negative affect likely had a partner who was also more reactive (*r *= .31).

### Gender Differences

We performed exploratory follow-up Wald chi-square tests to examine gender differences in conflict reactivity. For all outcomes, gender differences were insignificant, i.e., on average, female and male partners experienced conflict in similar ways. In a second step, we examined gender differences in all significant moderating effects. For negative affect, the moderating effect of relationship satisfaction on conflict reactivity was significant (*χ*^*2*^ [1, *n* = 151] = 4.80, *p* = .03), indicating that women low in relationship satisfaction were more negatively affected by conflict than men low in relationship satisfaction.

## Discussion

The present study examined the role of conflict on experiences of loneliness and affect in the daily lives of older intimate relationship partners in a naturalistic setting. To the best of our knowledge, only few studies have examined the role of daily relationship conflict on affective states in couples from other life stages, i.e., in middle adulthood (Almeida et al., 2002; Bolger et al., 1989). In these studies, conflict was assessed retrospectively and based on one daily observation. To our knowledge, reactivity to couple conflict in terms of loneliness has not yet been examined.

### Couple Conflict and Older Partners’ Experiences of Affect and Loneliness

Taken together, our findings align with prior studies on affect reactivity to conflict in everyday life (Almeida et al., 2002; Bolger et al., 1989). Similar to middle-aged couples, older intimate partners react with negative emotions, lack of positive emotions, or feelings of loneliness when confronted with a disagreement, argument, or conflict. Taking into account the subjective nature of loneliness (Ernst & Cacioppo, 1999) may explain increases in loneliness after conflict in old age: People experience loneliness when perceiving a discrepancy between the desired and actual state of relationship(s). In moments of conflict, this discrepancy may grow, and feelings of loneliness may arise. This is in line with prior research on loneliness in response to marital strain in older life stages (Chen & Feeley, 2014; Saenz, 2021). Importantly, older partners in our study experienced conflict in heterogeneous ways, in that some were likely to suffer more than others in response to it.

In terms of affective well-being in old age, our findings align with prominent theoretical accounts (e.g., Carstensen, 2006; Charles, 2010) as well as empirical research (Carstensen et al., 2011) suggesting that positive emotional experiences improve into the late 60s and level off thereafter. Similarly, loneliness has been found to unfold in a non-linear manner across the lifespan, with elevated levels of loneliness among the very old (Luhmann & Hawkley, 2016). While age was positively correlated with negative affect and loneliness in our study, age and positive affect showed negative associations (Table 1), indicating more negative affective experiences with advancing age. Older people in our study were likely challenged by age-related difficulties and problems, such as health impairments or diminishing social and emotional resources (Baltes & Smith, 2003; Heylen, 2010), leading to declining well-being in the oldest old.

Our results failed to show gender differences in reactivity to conflict. Although some lines of research assert that negative relational interactions, such as couple conflict, bear a more detrimental effect on women than on men in terms of health and well-being (see, Carr et al., 2016; Kiecolt-Glaser & Newton, 2001; Wanic & Kulik, 2011), findings on gender differences in loneliness in response to negative relational experiences or marital strain are more mixed (e.g., Hsieh & Hawkley, 2017; Saenz, 2021; Segel-Karpas & Ermer, 2021). A possible explanation for this absence of gender differences may be based on participants’ older age. Empirical research has shown that older couples generally try to avoid and refrain from relationship conflict (Birditt et al., 2005; Holley et al., 2013). Moreover, SAVI suggests that older adults respond with high affective reactivity or experiences of loneliness when they are unable to avoid or deescalate interpersonal conflict (Charles, 2010). Thereby, it is conceivable that disagreements, arguments and conflicts reported by participants in our study occurred in response to relatively serious topics and thus impacted both partners alike, with the exemption of comparatively higher reactivity in women with lower relationship satisfaction. Moreover, our findings align with prominent theories of lifespan gender development, such as Gutman’s (1987) theory of gender crossover, which posit that men and women become more alike with advancing age as they move on from stereotypes and duties attached to traditional marital roles (Lemaster et al., 2017). Thus, male and female partners in old age may be similarly affected by conflict.

### The Role of Moderators of Reactivity to Conflict

Another goal of our study was to disentangle risk and resilience factors associated with older intimate relationship partners’ reactivity to conflict in everyday life.

#### Individual-difference Characteristics

Among the most noteworthy of results was the extent to which neuroticism moderated reactivity to conflict for both women and men. Women and men high in neuroticism reported lower positive and higher negative affect overall, while men higher in neuroticism also reported higher levels of loneliness overall. Importantly, men high in neuroticism showed more reactivity in terms of increased loneliness after conflict, while women high in neuroticism were more reactive in terms of affect following conflict. These results are broadly consistent with and extend the existing literature on neuroticism and couple conflict. On the one hand, neuroticism is associated with more intense negative emotions (Kalokerinos et al., 2020) and decreased recovery (i.e., downregulation) following negative events (Suls et al., 1998). In addition, our results resonate with the assumption that appraisals of conflict interactions with partners are biased in individuals high in neuroticism. Individuals high in neuroticism may perceive partner behavior more negatively and be more reactive during and after conflict (Finn et al., 2013; McNulty, 2008). Especially in old age, this negativity bias may counteract attempts to avoid negative emotional experiences or evaluate spousal interactions positively and thus intensify negative reactivity.

Broadly consistent with prior studies (Lucas & Baird, 2004; Ng & Diener, 2009), extraversion predicted higher overall positive affect in men. However, we were unable to confirm buffering effects of extraversion on reactivity to conflict from previous research in middle adulthood (Almeida et al., 2002). Both studies differed in relevant methodological characteristics, such as participants’ age, affect measures (affect balance vs. positive and negative affect) and time scale (daily vs. momentary affect), which may account for discrepancy in findings.

#### Couple-level Factors

Consistent with previous research, lower relationship satisfaction related to higher overall loneliness in female as well as higher baseline negative affect in both partners (Røsand et al., 2012). It is plausible that older female partners grow increasingly lonely when they perceive their relationship as unsatisfactory. Lower relationship satisfaction increased negative affect following conflict for female, but not for male partners. Several explanations may be drawn upon to discuss this. First, unsatisfactory relationships are conceivably more deleterious for women, as they carry major responsibility for regulating the general emotional climate in the relationship (Bloch et al., 2014) and during specific couple interactions (Croyle & Waltz, 2002). This may be especially detrimental in old age, where spousal relationships are generally closer and increasingly characterized by meaningful emotional exchange (Antonucci, Ajrouch, & Birditt, 2014; Hoppmann & Gerstorf, 2009). Second, it has been argued that women desire affiliation more strongly than men in times of high distress (Taylor et al., 2000). When relationship quality is low and women are less able to affiliate, they may suffer more than men after stressful conflict episodes. Third and in line with this reasoning, studies have shown that higher relationship satisfaction resonates with increased compatibility of attitudes and goals within couples (Arránz Becker, 2013). Higher relationship satisfaction may foster goal compatibility and allow for more affiliation between partners, thus lowering female conflict reactivity. These explanations may seem contradictory to our earlier statements on gender crossover (Guttman, 1987). However, gender differences in terms of relationship satisfaction may have been even more pronounced when people were younger, thus still emerging in old age.

In contrast, high conflict frequency was related to women’s lower baseline positive and men’s higher baseline negative affect. Frequent fights are likely to bear a negative impact on partners general sentiments in daily life. For example, couples who experience frequent conflict may display more negative general sentiments towards each other, which are important contributors of the affective tone in which they interact (Hawkins et al., 2002) and likely render partners more apt to engage in frequent conflict in turn. Contrary to prior research (Almeida et al., 2002), conflict frequency was unrelated to partners’ affective reactivity in our study, which may be due to differences in relevant methodological and sampling characteristics, such as less measurement points and retrospective assessments of conflict, age of partners or relationship length. For example, participants in our study have upheld their relationships for four decades on average and are habituated to recurring numbers of conflict at this life stage. Participants in prior studies (e.g., Almeida et al., 2002; Bolger et al., 1989) were much younger on average and presumably exhibiting shorter relationship length on average. Based on this habituation, single arguments in our study may have been less likely to create a strenuous environment or threaten older partners’ sense of confidence that conflict can be weathered. As a result, older partners in more conflictual relationships may not respond with elevated arousal to every single argument.

### Strengths and Limitations

The current study expands on previous research by examining the role of conflict on older couples’ everyday experiences of affect and loneliness with the experience sampling method (ESM; Mehl et al., 2013). With up to 84 self-reports over 2 weeks, we were able to gather a detailed image on momentary conflict reactivity, and identified associated psychological risk and resilience factors. Reaching a better understanding of such factors will considerably aid interventions to improve individual well-being and dyadic relationship outcomes (Almeida et al., 2002).

Despite these strengths, several limitations to our study are to be noted. First, we focused on two typical weeks in the daily lives of our participants. Time periods which are atypical and highly stressful, i.e., severe illness or hospitalization of one partner, may provide more opportunity for conflict, and change partners’ reactivity patterns. Future research should address the role of relationship conflict under such conditions. Second, conflict behavior likely changes across adulthood and with increasing relationship duration (see, Kline et al., 2006). For example, younger adults may show higher rates of conflict over matters related to work and parenthood, whereas these topics may be less relevant in old age (Kline et al., 2006). Future studies should take an age-comparative approach to explore differences in conflict reactivity between younger and older couples, preferably with inclusion of relevant environmental factors. Third, the present study merely studied conflict in cross-sex couples. Future research should focus on more diverse sample with inclusion of same-sex couples to bolster the generalizability of results. Fourth, older couples typically have a longer relationship length and our sample included relatively few couples with a shorter relationship duration. However, couples with a high conflict frequency are likely to have terminated their relationship at this stage in life, and an average relationship length of multiple decades, as in our sample, may be taken as evidence of intensified resilience towards relationship obstacles and difficulties (Bühler et al., 2021). Beyond this, we only examined cohabiting/married couples. Future research should replicate our study across couples with less homogeneous relationship status and length.

As another important avenue of future research, the nature of conflict should be addressed in more detail. As Kline et al. (2006) point out, scholars have not unequivocally agreed upon a clear definition of conflict. Our study measured conflict as a dichotomous item, were participants indicated whether they experienced a disagreement, argument, or conflict with their partner since the last measurement occasion. This item may exhibit limited comparability across participants and measurement occasions. One the one hand, participants may differ in how they understand and interpret the specific content of this item (e.g., short argument vs. more severe conflict). On the other hand, the item lacks pertaining severity ratings indicating the extent to which participants experienced conflict as aversive and detrimental. In line with this, momentary conflict ratings (*r* = .47) as well as the conflict frequency across 2 weeks (*r* = .59) were highly but imperfectly correlated between partners. Partners may have answered questionnaires at different moments in time, i.e., before or after arguments, as they could delay questionnaires up to 4 times with a maximum delay time of 1 hour. On the other hand, partners may have interpreted arguments differently, with one partner perceiving a conflict and the other partner not perceiving an argument or conflict. Future studies should thereby control for partners’ interpretation of the unfolding conflict.

With respect to pursuing outcomes, patterns of severe conflict can unfold over long time periods, and some issues may be difficult to resolve and result in ongoing conflict (Caughlin et al., 2006). This can have more deleterious and long-lasting implications for people’s affective reactions than short-run disagreements. Adding to this, several lines of research support the notion that intimate partner’s underlying concerns during conflict, such as having one’s needs neglected or power threatened, are likely to trigger different reactions in intimate partners (Sanford & Grace, 2011). Some conflicts may threaten power and status of partners more than others, and it is indispensable to more precisely focus on the implications of unfolding conflict in future studies. In line with this, research has shown that not necessarily conflict itself, but the way in which couples deal with and communicate during conflict episodes impacts upon relationship quality and serves as a precursor for relationship dissolution (Kline et al., 2006). Future research should examine the role of conflict resolution and communication styles in reactivity to conflict.

With regard to the study outcomes, future ESM studies may address distinct emotions to better explore how they are affected by conflict episodes, as negative emotions such as sadness or anger are highly distinct and show multidirectional age differences (Kunzmann et al., 2014). For example, escalating anger may increase conflict, whereas sharing feelings of sadness may foster closeness and intimacy (Butler, 2011). In addition, loneliness was measured as a single item in the present study. Future research should target more facets of experiences of loneliness (de Jong-Gierveld & Kamphuls, 1985), such as feelings of being rejected or experiencing others as close to oneself and trustworthy.

## Conclusions

Our findings demonstrated that couple conflict likely leaves a negative mark on older adult’s everyday sentiments: Prior conflict increases experiences of loneliness and negative affect and decreases experiences of positive affect for female and male partners. The effect of conflict on emotional distress depends on individual difference characteristics of partners. Neuroticism is a risk factor exacerbating the detrimental effects of conflict on partners’ everyday feelings. On the contrary, relationship satisfaction may buffer reactivity to conflict for female partners. We conclude that relationship conflict can harm intimate partners’ feelings even in old age and after decades of spending life together as a couple. However, targeting risk factors and resources associated with conflict may improve individual well-being and dyadic relationship outcomes.

## Supplemental Material

Supplemental Material - The Role of Relationship Conflict for Momentary Loneliness and Affect in the Daily Lives of Older Couples Click here for additional data file. Supplemental Material for The Role of Relationship Conflict for Momentary Loneliness and Affect in the Daily Lives of Older Couples by Elisa Weber, and Gizem Hülür in Journal of Social and Personal Relationships
