# Supplementary material for: The Role of Relationship Conflict for Momentary Loneliness and Affect in the Daily Lives of Older Couples
Source: J Soc Pers Relat. 2022 Nov 10;40(7):2033–60. doi: 10.1177/02654075221138022 (PMC10333975; doi:10.1177/02654075221138022)
Supplement: Supplemental Material - The Role of Relationship Conflict for Momentary Loneliness and Affect in the Daily Lives of Older Couples [file sj-pdf-1-spr-10.1177_02654075221138022.pdf]

Supplemental Table 1. The Role of Conflict on Positive Affect Items (Baseline).

**Fixed Effects**	**Women**				**Men**			
	**Intercept**		**Conflict**		**Intercept**		**Conflict**	
Item	γ	SE	γ	SE	γ	SE	γ	SE
Happy	**75.14**	1.10	**–12.96**	1.37	**77.55**	1.08	**–9.92**	1.10
Content	**75.50**	1.04	**–14.93**	1.39	**77.45**	0.99	**–12.42**	1.30
Balanced	**68.81**	1.19	**–13.07**	1.28	**71.77**	1.06	**–10.45**	1.15
Inspired	**67.31**	1.25	**–4.66**	1.15	**66.82**	1.37	**–3.94**	0.76

Notes. n = 151 couples. γ = regression coefficient; SE = standard error. Fixed effects = regression coefficients and standard errors are presented. Outcome variable: Positive affect items on a scale from 0% (not at all) to 100% (very much). *Statistical* significance evaluated at $p < 0.05$. Significant p-values in bold.

Supplemental Table 2. The Role of Conflict on Negative Affect Items (Baseline).

**Fixed Effects**	**Women**				**Men**			
	**Intercept**		**Conflict**		**Intercept**		**Conflict**	
Item	γ	SE	γ	SE	γ	SE	γ	SE
Nervous	**22.51**	1.31	**9.38**	1.23	**20.75**	1.19	**8.93**	1.34
Sad	**12.56**	1.11	**8.32**	1.35	**10.60**	0.88	**5.71**	1.09
Anxious	**21.73**	1.39	**5.86**	1.19	**19.14**	1.30	**7.12**	1.08
Upset	**14.50**	0.96	**17.41**	1.76	**13.74**	0.96	**13.94**	1.48

Notes. n = 151 couples. γ = regression coefficient; SE = standard error. Fixed effects = regression coefficients and standard errors are presented. Outcome variable: Negative affect items on a scale from 0% (not at all) to 100% (very much). *Statistical* significance evaluated at $p < 0.05$. Significant p-values in bold.

Supplemental Table 3. The Role of Conflict on Experiences of Loneliness (Baseline) with Exclusion of Cohabiting Couples.

**Fixed Effects**						
	**Women**				**Men**	
Predictor	γ		SE		γ	SE
Intercept	**13.04**		1.17		**12.55**	0.94
Conflict	**5.58**		1.33		**6.56**	1.35
**Random Effects**						
	**Variance (σ^2)**	**Correlation**				
		(1)	(2)	(3)	(4)	
Women:Intercept (u_{0iw}) (1)	185.0					
Men:Intercept (u_{0im}) (2)	113.5	0.53				
Women: Conflict (u_{1iw}) (3)	112.9	0.15	0.08			
Men:Conflict (u_{1im}) (4)	112.4	0.00	0.03	0.45		
Residual	260.9					

Notes. n = 134 couples. w = women, m = men. γ = regression coefficient; SE = standard error; u = random effect; σ^2 = variance. Fixed effects = regression coefficients and standard errors are presented. Random effects = variances and correlations are presented. Outcome variable: loneliness on a scale from 0% (not at all) to 100% (very much). *Statistical* significance evaluated at $p < 0.05$. Significant p-values in bold.

Supplemental Table 4. The Role of Conflict on Experiences of Positive Affect (Baseline) with Exclusion of Cohabiting Couples.

**Fixed Effects**						
	**Women**				**Men**	
Predictor	γ		SE		γ	SE
Intercept	**71.85**		1.04		**73.30**	0.97
Conflict	**–10.94**		1.22		**–9.47**	0.95
**Random Effects**						
	**Variance (σ^2)**	**Correlation**				
		(1)	(2)	(3)	(4)	
Women:Intercept (u_{0iw}) (1)	142.6					
Men:Intercept (u_{0im}) (2)	125.3	0.38				
Women: Conflict (u_{1iw}) (3)	123.7	–0.15	–0.14			
Men:Conflict (u_{1im}) (4)	59.29	–0.14	0.00	0.46		
Residual	117.3					

Notes. n = 134 couples. w = women, m = men. γ = regression coefficient; SE = standard error; u = random effect; σ^2 = variance. Fixed effects = regression coefficients and standard errors are presented. Random effects = variances and correlations are presented. Outcome variable: positive affect on a scale from 0% (not at all) to 100% (very much). *Statistical* significance evaluated at $p < 0.05$. Significant p-values in bold.

Supplemental Table 5. The Role of Conflict on Experiences of Negative Affect (Baseline) with Exclusion of Cohabiting Couples.

**Fixed Effects**					
	**Women**			**Men**	
Predictor	γ	SE		γ	SE
Intercept	**17.37**	1.07		**16.10**	0.98
Conflict	**10.16**	1.17		**8.65**	1.08

**Random Effects**					
	**Variance** (σ^2)	**Correlation**			
		(1)	(2)	(3)	(4)
Women:Intercept (u_{0iw}) (1)	151.16				
Men:Intercept (u_{0im}) (2)	126.94	0.48			
Women: Conflict (u_{1iw}) (3)	110.64	–0.08	–0.08		
Men:Conflict (u_{1im}) (4)	82.16	–0.02	0.17	0.37	
Residual	121.41				

Notes. n = 134 couples. w = women, m = men. γ = regression coefficient; SE = standard error; u = random effect; σ^2 = variance. Fixed effects = regression coefficients and standard errors are presented. Random effects = variances and correlations are presented. Outcome variable: negative affect on a scale from 0% (not at all) to 100% (very much). *Statistical* significance evaluated at $p < 0.05$. Significant p-values in bold.
